# Transmission Line-Planning Method Based on Adaptive Resolution Grid and Improved Dijkstra Algorithm

**DOI:** 10.3390/s23136214

**Published:** 2023-07-07

**Authors:** Guojun Nan, Zhuo Liu, Haibo Du, Wenwu Zhu, Shuiqing Xu

**Affiliations:** School of Electrical Engineering and Automation, Hefei University of Technology, Hefei 230000, China2021110428@mail.hfut.edu.cn (Z.L.);

**Keywords:** Dijkstra algorithm, ground object-identification image, adaptive resolution grid, bidirectional search, inflection point-correction

## Abstract

An improved Dijkstra algorithm based on adaptive resolution grid (ARG) is proposed to assist manual transmission line planning, shorten the construction period and achieve lower cost and higher efficiency of line selection. Firstly, the semantic segmentation network is used to change the remote sensing image into a ground object-identification image and the grayscale image of the ground object-identification image is rasterized. The ARG map model is introduced to greatly reduce the number of redundant grids, which can effectively reduce the time required to traverse the grids. Then, the Dijkstra algorithm is combined with the ARG and the neighborhood structure of the grid is a multi-center neighborhood. An improved method of bidirectional search mechanism based on ARG and inflection point-correction is adopted to greatly increase the running speed. The inflection point-correction reduces the number of inflection points and reduces the cost. Finally, according to the results of the search, the lowest-cost transmission line is determined. The experimental results show that this method aids manual planning by providing a route for reference, improving planning efficiency while shortening the duration, and reducing the time spent on algorithm debugging. Compared with the comparison algorithm, this method is faster in running speed and better in cost saving and has a broader application prospect.

## 1. Introduction

As an important part of the power-transmission and -transformation project, route selection of transmission lines is related to the planning and construction of an entire power grid and has a profound impact [1] on the economic development and residents’ life in the local area. The traditional route-selection method involves a large workload and a long construction period and its main disadvantage is that human factors have a great influence. In the face of the increasingly complex scale of the power grid, digital design is widely used in various fields and it is difficult to meet the higher demand of power grid construction by using traditional artificial transmission line planning [2]. Using a computer [3] to select the transmission line routes with an intelligent algorithm can significantly shorten the construction period, and using scientific and reasonable layout planning is of great significance to power-transmission and -transformation projects.

The quality of transmission line planning [4] is directly related to geographical environment [5,6], social influence, actual construction cost, operation, maintenance and other factors. The environments of areas through which power lines pass are relatively complex. Different areas, such as forests, rivers, mud and residential areas, cause different costs. The terrain types in the planning area are obtained and classified via geographic information system (GIS) technology [7] and the unit cost value contained in each grid is obtained via the fuzzy analytic hierarchy process. The traditional shortest path search algorithms include particle swarm optimization [8,9], ant colony algorithm [10,11,12], A-star algorithm [13], etc. When these algorithms are used for large-scale maps, the running time is very long, it is not easy to obtain the global optimal solution and the line selection is not ideal. The Dijkstra algorithm [14,15] does not require iteration and takes a short time. As long as there is an optimal route, it can be found, and it is suitable for line-selection problems involving factors such as route length, number of corners and construction costs in different environments.

In this paper, we use semantic segmentation to change the remote sensing image into a ground object-identification image [16,17] that retains the geographic environment information affecting the construction of transmission lines. By rasterizing the grayscale image of the ground object-identification image and establishing an ARG map cost model, the large number of redundant grids is reduced and the efficiency of line selection is improved. Based on the Dijkstra algorithm, the bidirectional search mechanism is adopted to make the algorithm more efficient in route searches in large-scale planning areas. A multi-center grid neighborhood structure is used to suppress line distortion. Then the inflection point-correction mechanism is introduced to smooth the route distortion and reduce the cost of line selection. After experimental verification, the bidirectional Dijkstra algorithm based on ARG has higher line-selection efficiency than the basic Dijkstra algorithm-based single resolution grid (SRG) and can obtain lower cost lines.

## 2. Adaptive Resolution Grid Map Modelization

Transmission line planning is actually path planning based on grid maps. Firstly, the feature extraction of a remote sensing image using the Deeplabv3+ semantic segmentation network is used to obtain the ground object-identification image, and is then converted to a grayscale image and the grayscale image is adaptively segmented to obtain the ARG map. Multiple influencing factors affecting transmission line planning are quantified and then the cost value of each grid is obtained via the fuzzy hierarchical process to establish the ARG map model.

### 2.1. Remote Sensing Image Segmentation of Ground Objects

In this paper, the Deeplabv3+ deep learning model is used to extract ground elements in remote sensing images that affect transmission line planning, so that some key ground elements can be easily distinguished and interpreted from other ground objects.

**Figure 1:**
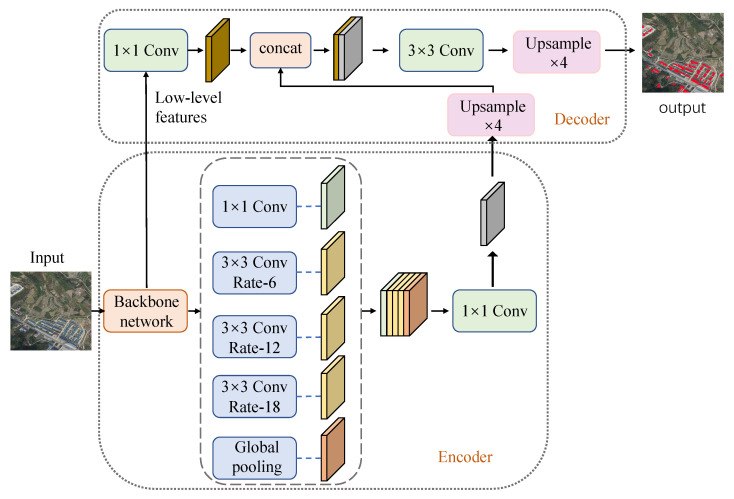
Deeplabv3+ network structure.

Deeplabv3+ uses an encoding–decoding structure, the encoder extracts high-level semantic features [18] and the decoder uses bilinear interpolation to perform upsampling to gradually restore boundary information. The algorithm structure is shown in Figure 1 can be outlined as follows.

Encoder: The deep convolutional neural network performs feature extraction on the input image and then strengthens the feature-extraction ability through the ASPP structure. The ASPP module performs 1×1 convolution, 3×3 convolution with expansion rates of 6, 12 and 18 and global average pooling operations on the feature layers passing through the backbone network. After concat, 1×1 convolution is used to compress the number of channels to 256. The parallel structure of ASPP can extract feature information of different scales and improve the segmentation effect of multi-scale targets.

Decoder: The extracted high-level semantic features are upsampled to make up for the boundary information lost in the downsampling process. In the process of feature map restoration, the low-level features are merged and the high-level features are further supplemented by the richer texture information contained in the low-level features [19]. Finally, through four-fold linear upsampling, the final prediction image is obtained.

The final predicted image is the desired ground object-identification image.

### 2.2. Introduction of Single Resolution Grid Map

The format of data stored in GIS is mainly raster data and vector data [20,21,22]. GIS-based vector data structures are common in network problems based on lines, edges and nodes. However, the actual terrain information is not suitable for associating with nodes and lines, but can be associated with polygons or small areas of basic cells. The map is divided into grid cells for the subsequent route search. So the grid data are used to build a grid map for path planning.

The SRG map [23] is composed of regular squares and each square unit grid represents a basic area. The accuracy of geospatial information segmentation depends on the size of the basic cell grid, i.e., the size of the resolution (e.g., a cell with a resolution of 50×50 represents 50×50 pixels). A grid contains the cost of passing a transmission line through the grid [24,25], which is directly related to factors such as the social impact of overhead line planning, actual construction costs, operation, maintenance and the geographic location of the grid in the map.

### 2.3. Adaptive Quadtree Image Segmentation

The traditional image single-resolution rasterization method cannot solve the problem of reducing the number of grids and reducing the fitting error (the degree of dissimilarity between the rasterized image and the original image ). In extreme cases, when the size of the pixel block (grid) in the whole image is close to the size of the original remote sensing image, the requirement of a small number of grids is satisfied, but the fitting error is large, resulting in the problem of insufficient image-processing accuracy. In areas with diverse geographical environments, grids with the same resolution as other single terrain areas are used, which will make the planned path position accuracy low. In contrast, when the size of the pixel block approaches the size of the image pixel, the color distribution and terrain edge features of the rasterized image approach the original image and meet the requirement of minimal fitting error. However, the number of segmented grids and the amount of data are redundant, resulting in long calculation time and low efficiency in the application of the next path-planning algorithm.

Therefore, based on the quadtree structure [26,27,28,29], this paper proposes an adaptive quadtree image-segmentation [30,31,32] method, which can simultaneously segment the remote sensing images with different characteristics to obtain the information of each object, the edge of the unit grid object, the gray level of the object, the size of the object, etc., forming a grid map with accurate edge of the object. It also meets the needs of reducing the number of grids and reducing the fitting error. The mean value of each pixel block is obtained by using as few grids as possible to complete the optimal fitting of the original remote sensing image, so that the Dijkstra algorithm can be used to plan the transmission line path of the grid map containing various components.

In the adaptive segmentation algorithm, the object-identification map of the remote sensing image is first converted into a grayscale image and then the grayscale image is adaptively segmented. The maximum grid size is 16 times the minimum grid size. The maximum grid size is the resolution of the initial rasterization of the grayscale image. Key elements in partition include the following:

- The grayscale variance of grid block;
- The minimum grid size.

The segmentation judgment condition (SJC) refers to the basis for whether the current grid is segmented by the quadtree. The SJC is divided into two parts.

(1) If the size of the current grid is not greater than the minimum grid size, the current grid is not segmented; otherwise, the second judgment condition is performed.

(2) If the variance of the gray value of the current grid is greater than the variance threshold $V_{f}$, segmentation is needed. Otherwise, it is judged whether the variances of the gray value of the 4×4 small grids in the grid are less than the mean threshold $V_{s}$. If so, the current grid is not segmented; otherwise, the segmentation continues.

In order to meet sufficient reliability requirements, $V_{f}$ should be set less than 160 and $V_{s}$ should generally be set less than 16.

**Figure 2:**
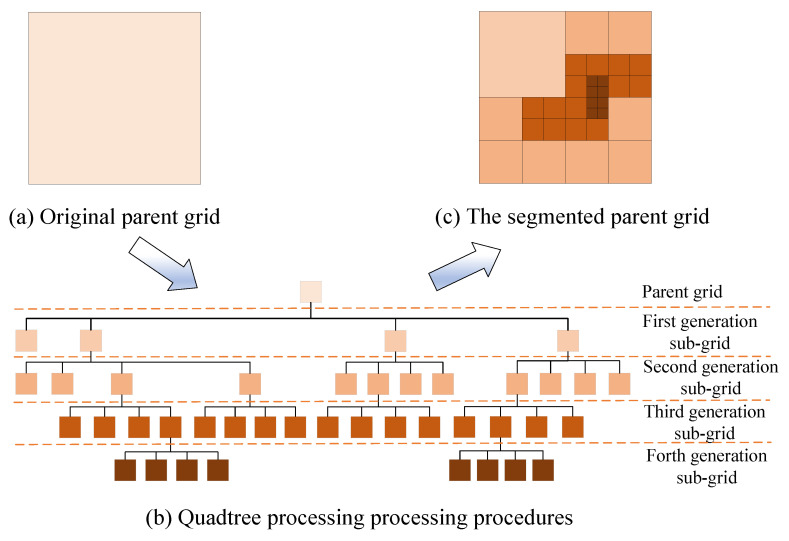
Segmentation process of parent grid.

The segmentation process of the adaptive quadtree image-segmentation method is shown in Figure 2. Firstly, the original remote sensing image is rasterized with a single resolution. The grid resolution is determined by the maximum grid size and the grid unit at this time is called the parent grid. Next, the first grid segmentation is started. If the SJC is satisfied, the segmentation generates four sub-grids, which are called the first generation sub-grids (R1, R2, R3, R4). Then the second-round grid segmentation is performed and the sub-grid units that meet the conditions are called second-generation sub-grids. By analogy, each grid of the entire image no longer meets the SJC. Finally, a complete ARG map is formed.

The number of pixels in the grid block *G* is recorded as *N* and the gray mean $\bar{\chi }$ and gray variance $\sigma^{2}$ of the grid are generated by

(1) $$\begin{array}{cc} \bar{\chi } & =\frac{1}{N}\sum_{(x,y)\in G}f\left(x,y\right), \end{array}$$

(2) $$\begin{array}{cc} \sigma^{2} & =\sum_{(x,y)\in G}{\left(f\left(x,y\right)-\bar{\chi }\right)}^{2}, \end{array}$$

where (x,y) is the coordinate of the pixel and f(x,y) is the gray value of the pixel.

**Figure 3:**
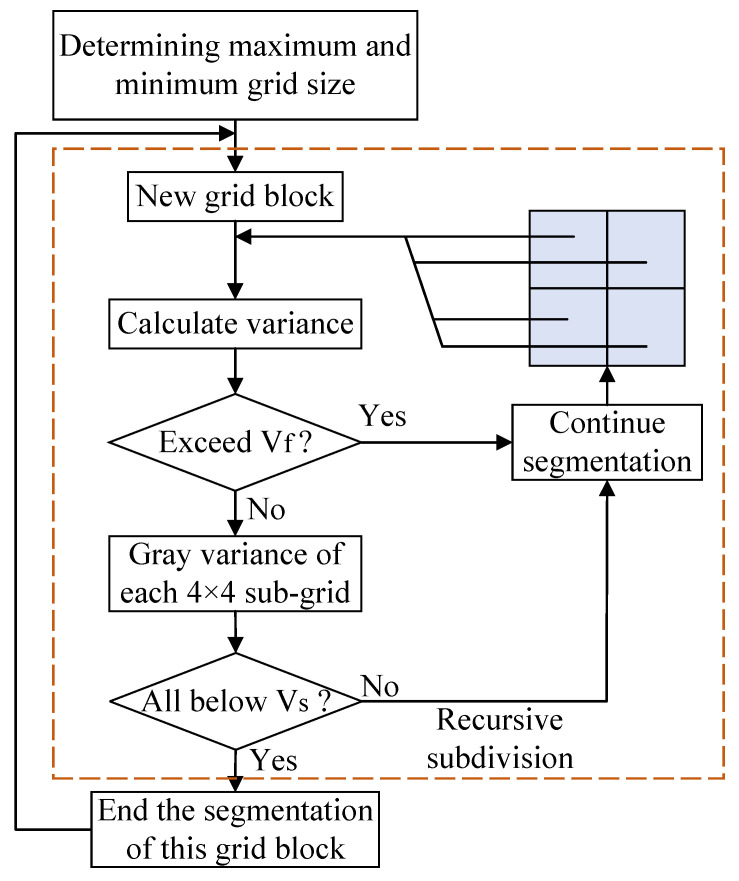
Adaptive quadtree image-segmentation process.

The adaptive quadtree image-segmentation process is shown in Figure 3.

**Figure 4:**
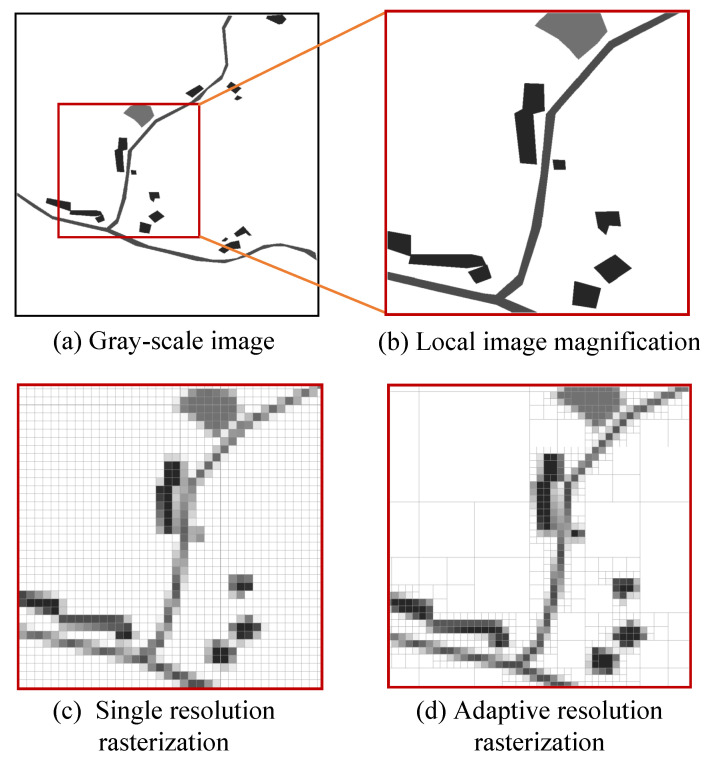
Comparison of SRG and ARG.

The gray image of a ground object-identification image is rasterized with single resolution and adaptive resolution, respectively. A schematic is shown in Figure 4.

### 2.4. Adaptive Resolution Grid Cost Calculation

Each grid contains the economic cost required for the transmission line to pass through this area. This paper simplifies various factors affecting this cost. The cost is related to the construction cost, wire cost and social impact cost in this area. These three factors directly affect the pros and cons of transmission line planning.

**Figure 5:**
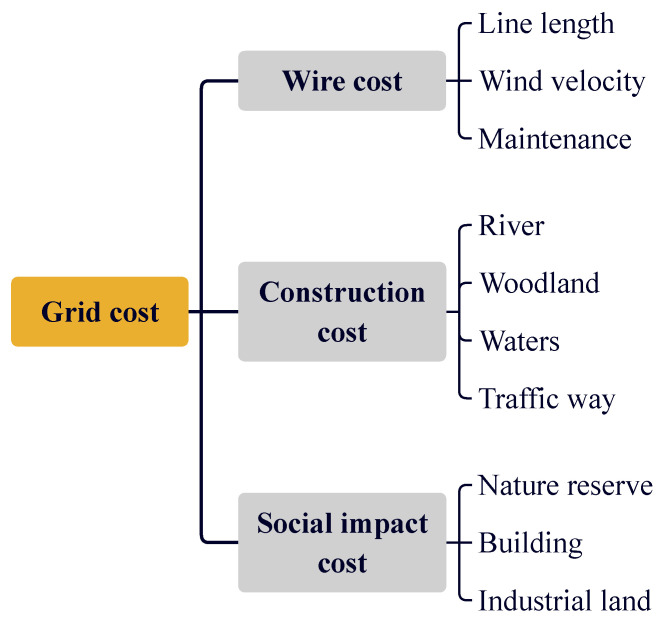
Hierarchical structural drawing of grid cost.

The three kinds of influence factors of line selection are divided into several evaluation indexes by class. The hierarchical structural drawing of Figure 5 is divided into three levels, which are subdivided into target layer, criterion layer and index layer.

We quantify the rating scale of the evaluation indexes $s=\left[s_{1},s_{2},\cdots ,s_{10}\right]$. The rating scale is indicated by 1–5, where 1 indicates that the current environment is very suitable for the construction of transmission lines and the cost is very low, and 5 indicates that the current environment is very unsuitable for the construction of transmission lines and the cost is very high. Then combined with the fuzzy analytic hierarchy process [33,34,35], the comprehensive weight of each evaluation index for the grid cost $\omega ={\left[\omega_{1},\omega_{2},\cdots ,\omega_{10}\right]}^{T}$ is obtained. The cost value of the grid *c* is obtained as follows:

(3) c=s·ω.

## 3. Dijkstra Algorithm and Improvement

In this paper, the Dijkstra algorithm is combined with ARG and bidirectional search is used to further accelerate the efficiency of transmission line planning. The neighborhood structure of the grid adopts a multi-center neighborhood structure and inflection point-correction is performed on the planned line to remove the excess inflection points.

### 3.1. Dijkstra Algorithm

The Dijkstra algorithm is a greedy algorithm used to solve the single-source shortest path problem. Its purpose is to find the shortest path from the source node to the target node in a weighted directed graph (or undirected graph). It is widely used in routing algorithms and other network applications.

The basic idea of the algorithm is to establish a distance array from the source node to record the shortest distance from the current node to the source node. Then, starting from the source node, according to the order of the distance between the nodes in the distance array, the adjacent nodes are traversed from the node with the smallest distance to update the value of the corresponding node in the distance array. This process will continue until all nodes are traversed.

When the Dijkstra algorithm is applied to the grid map, the grid is used instead of the node and the distance array is changed into the cost array. The cost array is used to store the cumulative minimum route cost from the starting grid to the current grid. The corresponding value of the starting and ending grids in the cost array is 0 and the cost value in the cost array corresponding to the untraversed grid is positive infinity. The cumulative minimum route cost of the *i*th (i> 1) grid $c_{al}^{i}$ is:

(4) $$c_{al}^{i}=\left\{\begin{array}{cc} c_{al}^{j}+c_{i}\cdot l_{i}+q, & \;\mathrm{if}\;\mathrm{there}\;\mathrm{is}\;\mathrm{an}\;\mathrm{inflection}\;\mathrm{point},\;\\ c_{al}^{j}+c_{i}\cdot l_{i}, & \;\mathrm{if}\;\mathrm{there}\;\mathrm{is}\;\mathrm{no}\;\mathrm{inflection}\;\mathrm{point},\; \end{array}\right.$$

where $c_{al}^{j}$ is the cumulative minimum route cost of the previous grid *j* of the *i*th grid, $c_{i}$ is the grid cost of the *i*th grid, $l_{i}$ is the length of the line within the *i*th grid and *q* is the weight of the inflexion point. If a corner (i.e., an inflection point) is generated between the line between grid *i* and grid *j* and the previous line of grid *j*, the first calculation method of (4) is adopted; otherwise, the second calculation method of (4) is adopted.

### 3.2. Combination of Dijkstra Algorithm and Adaptive Resolution Grid

The Dijkstra algorithm used in route planning is based on SRG. The grid neighborhood structure of SRG is the single-center neighborhood structure. When the rasterized remote sensing image uses the SRG, the smaller the grid, the better the fit of the remote sensing image, and the distortion of the route will be very slight. However, at the same time, the running time of the program will be longer. If a heuristic search algorithm requiring iterations is used, the running time will be very long. Increasing the grid size will reduce the running time of the program, but will make the distortion of the route based on the single-center neighborhood structure severe. That is, the route mapped on the grid image to the remote sensing image will have an unreasonable location of the route. The size of the neighborhood grid of each grid in the ARG is uncertain, resulting in a large size of the grid in the neighborhood. The mobile direction during path planning will be significantly reduced, exacerbating the distortion of the route. However, when the ARG is combined with the multi-center neighborhood structure, it not only shortens the program running time but also improves the route distortion by increasing the optional directions during route search.

**Figure 6:**
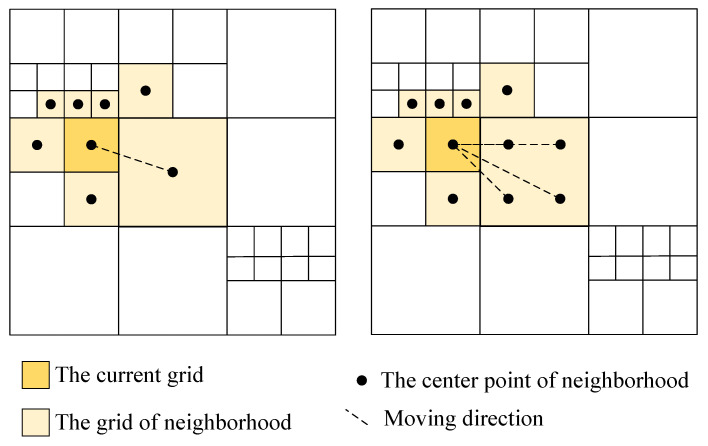
Multi-center neighborhood structure.

As shown in Figure 6, when there are four center points in the neighborhood grid, the optional moving direction is more than the single center point. The rule for determining the number of center points is that one center point is placed in the grid with a size of 1 or 2 times the minimum grid size, four center points are placed in the grid with a size multiple of 4 or 8, and 16 center points are placed in the grid with the maximum size (due to space limitations, not shown in Figure 6). The multi-center neighborhood structure can not only increase the direction of movement during route planning, effectively suppress route distortion and make it easier to obtain a better route, but also will not produce a large number of center points, increasing the amount of calculation and slowing down the speed of the program.

### 3.3. Bidirectional Search and Inflection Point-Correction Mechanism

In route planning, the Dijkstra algorithm has faster speed than the algorithm that needs to be iterated. However, as the scale of the map expands, the number of grids also increases and the search efficiency will be greatly reduced. Therefore, a bidirectional search mechanism based on ARG is introduced; that is, the forward and reverse bidirectional grid traversal is performed from the starting point and the end point, respectively, and the starting point of one is set as the endpoint of the other.

**Figure 7:**
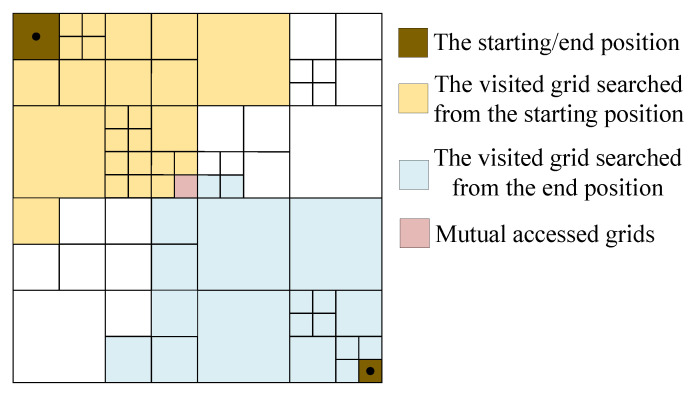
Bidirectional search of Dijkstra algorithm.

Two cost arrays Cs, Ce are created from the start and end positions, respectively, and the values are updated according to the size of the values in Cs and Ce. The visited sets Ts, Te are created to record the visited grids. Forward search and reverse search are carried out simultaneously, and every time a grid is visited, it is recorded in Ts, Te, and Cs, Ce is updated, which represents the completion of a bidirectional search step. For each step of the two-way search, we determine whether $T_{s}\cap T_{e}\neq ⌀$ is satisfied, i.e., whether the forward and reverse search process has produced a commonly visited grid. If yes, the route from the starting point to the current grid and the route from the endpoint to the current grid are extracted and then merged to get the complete route; otherwise, a new round of the search process is continued. The solid line in Figure 7 is the desired route.

The bidirectional Dijkstra search method greatly reduces the range of searches required and reduces the time consumed.

**Figure 8:**
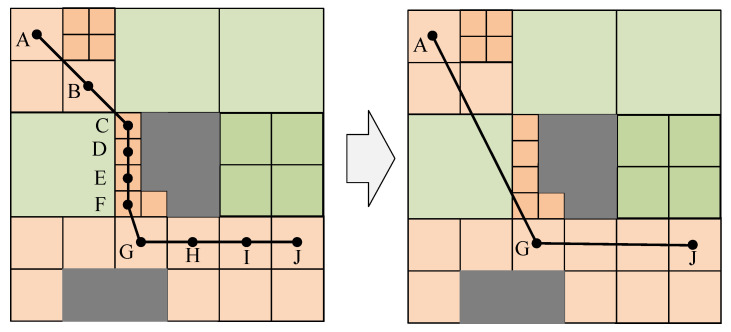
The inflection point-correction mechanism.

After the route search is completed, there will be more inflection points in the route. The corner tower must be established where there is an inflection point in the transmission line and the cost of establishing the corner tower accounts for a high proportion of the cost of the whole line planning. The extra inflection points will cause unnecessary cost expenditure. So reducing the number of inflection points is particularly important. Figure 8 shows the process of inflection point-correction, in which the gray part represents the area that the transmission line cannot cross.

The inflection point-correction principle is as follows: in the line of three points connected, if the two-endpoint-connected line route cost is lower than the original route, remove the center point and connect the two endpoints; otherwise, otherwise no change. The cost of non-crossing areas is infinite. In Figure 8, A, B and C are collinear and the middle point B is removed and then A and C are connected. The slope of the line segment AC is inconsistent with that of CD. AD does not pass through the non-crossing area. The route cost of AD is lower than the sum of the route cost of AC plus CD plus the corner tower cost; that is, the intermediate point C can be removed and the points A and D can be connected. The slope of line segment AG is different from that of GH, but AH crosses the gray area, so point G cannot be removed, and so on until the last point of the route is detected.

The route cost after inflection point-correction is calculated according to Equation (4).

## 4. Experiment and Discussion

### 4.1. Experimental Results

We selected two remote sensing images located in the Anhui Province of China for experimental simulation. The *q* value is set as q=1000.

**Figure 9:**
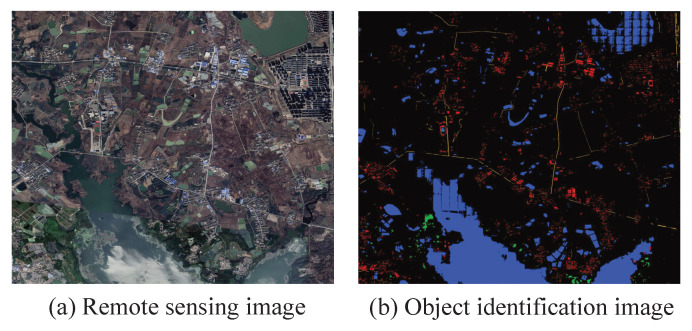
Test image T1.

The actual range of remote sensing image T1 in Figure 9 is 4.3 km × 5 km. In the ground object-identification map, blue represents water, red represents houses, yellow represents roads and green represents woodland. Set the red part as an area the transmission line cannot cross.

**Figure 10:**
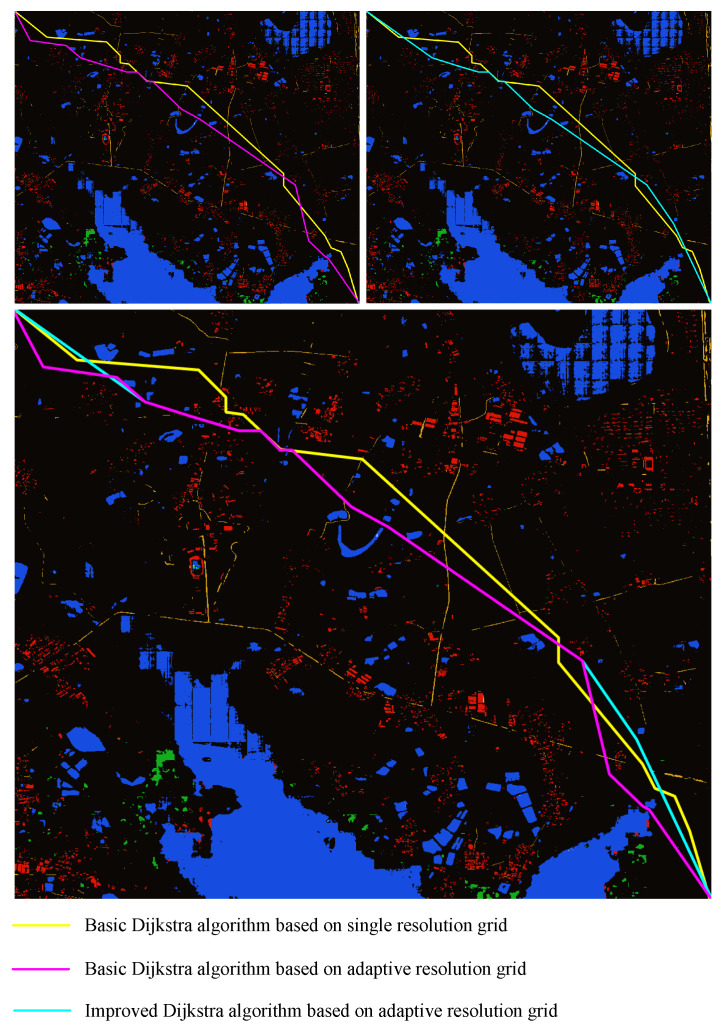
Line comparison of T1.

The grid size of the image T1 single-resolution rasterization is 70×70 and the corresponding actual range is about 20 m × 20 m. The minimum grid size of ARG is 70×70. In Figure 10, the yellow line is obtained via the basic Dijkstra algorithm based on SRG, the purple line is obtained via the basic Dijkstra algorithm based on ARG and the blue line is obtained via the improved Dijkstra algorithm based on ARG. The ARG in the experiment uses the multi-center neighborhood structure and the SRG uses the single-center neighborhood structure.

Table 1 shows the data comparison of the three lines. Combined with the line comparison in Figure 10 and the data in Table 1, it can be seen that when using T1 as the test map and using the basic Dijkstra algorithm, the planning speed of the algorithm based on ARG is 56.2% higher than that of SRG. Comparing the data of line length, cost value and inflection point number shows that the effect of line planning based on ARG is similar to that based on SRG. When the improved Dijkstra algorithm based on ARG is compared with the basic Dijkstra algorithm, the planning speed is increased by 69%, the line length is reduced by 2.3%, the cost value is reduced by 6.9% and the number of inflection points is reduced by 28.6%. According to the information in Table 2, after rasterizing the image T1, the number of grids using ARG is 53.1% less than that of SRG.

### 4.2. Discussion

From the experimental results, the basic Dijkstra algorithm based on ARG is indeed inferior to the one based on SRG in terms of the final cost value. So the experiment also used the improved Dijkstra algorithm based on ARG to participate in the comparison. The improved Dijkstra algorithm based on ARG outperforms the basic Dijkstra algorithm based on SRG in terms of both running time and cost value. In this experiment, the running time difference before and after the algorithm improvement is not large, but the time difference becomes large when the scope of the sensing image is expanded or when an algorithm requiring iterative convergence is used. The method proposed in this research not only reduces the running time but also provides a new option for reducing the time used in other path-planning fields.

## 5. Conclusions

To improve the efficiency of transmission line planning and obtain better results, this paper presents an improved Dijkstra algorithm based on ARG. The improved Dijkstra algorithm is a bidirectional search added to the basic Dijkstra algorithm and the bidirectional Dijkstra algorithm is applied to the ARG incorporating the multi-center neighborhood structure. The improved algorithm uses the inflection point-correction mechanism. According to the experimental results, the conclusions are as follows:

(1) In transmission line planning, the improved Dijkstra algorithm has higher operating efficiency than the basic Dijkstra algorithm. The final inflection point-correction of the algorithm reduces the number of corner towers that need to be established.

(2) After combining the improved Dijkstra algorithm with the ARG, the operation efficiency is further improved by comparing the results of the SRG operation and can obtain a similar line-selection effect.

(3) Compared with the basic Dijkstra algorithm, the proposed method effectively shortens the planning time of transmission lines and saves time cost. In the actual project, because this method takes into account the factors of line length and corner towers that affect the line selection, the planned line reduces more costs, has more reference value for operators and has broad application prospects.

## Figures and Tables

**Table 1:** Data comparison of case 1.

	Basic Dijkstra Algorithm Based on SRG	Basic Dijkstra Algorithm Based on ARG	Improved Dijkstra Algorithm Based on ARG
Running time (s)	641	281	87
Line length (m)	7061	6975	6812
Value of cost	61,817.89	65,024.72	60,521.75
Number of inflection points	14	14	10

**Table 2:** The number of grids of T1.

	SRG	ARG
Number of grids	67,920	31,845

## Data Availability

The data used to support the findings of this study are available from the corresponding author upon request.
